# Attenuation of Bleomycin-Induced Pulmonary Fibrosis in Rats with *S*-Allyl Cysteine

**DOI:** 10.3390/molecules22040543

**Published:** 2017-03-29

**Authors:** Takuma Tsukioka, Shigekazu Takemura, Yukiko Minamiyama, Shinjiro Mizuguchi, Michihito Toda, Shigeru Okada

**Affiliations:** 1 Department of Thoracic Surgery, Osaka City University Graduate School of Medicine, Osaka 5458585, Japan; m1156870@med.osaka-cu.ac.jp (T.T.); m1293795@msic.med.osaka-cu.ac.jp (S.M.); m_toda@hotmail.co.jp (M.T.); 2 Department of Hepato-Biliary-Pancreatic Surgery, Osaka City University Graduate School of Medicine, Osaka 5458585, Japan; yukiko-m@kpu.ac.jp; 3 Food Hygiene and Environmental Health Division of Applied Life Science, Graduate School of Life and Environmental Sciences, Kyoto Prefectural University, Sakyo-ku, Kyoto 6068522, Japan; 4 Graduate School of Medicine, Dentistry and Pharmaceutical Sciences, Okayama University, Okayama 7008558, Japan; dragon40@beach.ocn.ne.jp

**Keywords:** *S*-allyl cysteine, bleomycin, lung fibrosis

## Abstract

Pulmonary fibrosis is a complex disease with high mortality and morbidity. As there are currently no effective treatments, development of new strategies is essential for improving therapeutic outcomes. *S*-allyl cysteine (SAC) is a constituent of aged garlic extract that has demonstrated efficacy as an antioxidant and anti-inflammatory agent. The current study examines the effects of SAC on pulmonary fibrosis induced by a single intratracheal instillation of bleomycin (2.5 mg/kg). SAC was administered to rats as 0.15% SAC-containing diet from seven days prior to instillation up until the conclusion of the experiment (14 days post-instillation). SAC significantly reduced collagen mRNA expression and protein deposition (33.3 ± 2.7 μg/mg and 28.2 ± 2.1 μg/mg tissue in vehicle- and SAC-treated rats, respectively), and decreased fibrotic area, as assessed histologically. In the rats’ lungs, SAC also attenuated the increased expression of transforming growth factor-β1 (TGF-β1), a central regulator of myofibroblast recruitment, activation, and differentiation. While bleomycin instillation increased the number of myofibroblasts within the lung mesenchymal area, this change was significantly reduced by SAC treatment. SAC may exert efficacy as an anti-fibrotic by attenuating myofibroblast differentiation through TGF-β1-mediated fibroproliferative processes. Thus, our results indicate SAC may be useful for the prevention or treatment of pulmonary fibrosis.

## 1. Introduction

Pulmonary fibrosis is a complex disease with high mortality and morbidity [1]. Pulmonary fibrosis is characterized by the infiltration of inflammatory cells, the generation of reactive oxygen species, fibroblast proliferation, and the deposition of extracellular matrix protein into the lung parenchyma. Anti-inflammatory drugs such as corticosteroids and immunosuppressive agents are widely used for pulmonary fibrosis treatment; however, there is no truly effective treatment for this disease [2,3,4]. Therefore, the development of new agents is essential for improving therapeutic efficacy. Recently, pirfenidone treatment has been reported to provide a means of intervention for the clinical course of idiopathic pulmonary fibrosis (IPF) [5]. Though this is a promising candidate for improving patient prognosis, the authors note the importance of establishing appropriate treatment modalities for pirfenidone and/or other novel drugs in the future.

Used as a traditional medicine for centuries, garlic has been scientifically shown to prevent inflammation and thrombosis and reduce cellular oxidative damage [6]. However, the effective molecules within the composition of garlic and their pharmacological behaviors have not been clearly determined. *S*-allyl cysteine (SAC) is an odorless and stable constituent of aged garlic extract with low toxicity. Amagase et al. reported in their pharmacokinetic study that SAC was easily absorbed from the gastrointestinal tract and distributed into the plasma, liver, kidneys, lungs, and heart, with a bioavailability of 98% in rats [6]. Several reports have described the biological effects of SAC in various organs. For example, SAC reduced ischemic brain edema in rats by inhibiting lipid peroxidation [7], and reduced histological damage to the heart in mice treated with doxorubicin, an anticancer drug [8]. We previously reported that SAC decreased carbon tetrachloride (CCl_4_)-induced acute liver injury by attenuating oxidative stress [9] and liver fibrosis [10].

Numerous cytokines and growth factors have been implicated as mediators in the pathogenesis of pulmonary fibrosis. One such mediator, transforming growth factor β1 (TGF-β1), is a key regulator of both normal wound repair and the aberrant repair mechanisms characteristic of many fibrotic diseases, including pulmonary fibrosis [11]. It is believed that TGF-β1 is a central regulator of the recruitment, activation, and differentiation of myofibroblasts during the early phases of tissue repair [12]. The infiltration of the myofibroblastic phenotype in areas of active fibrosis is a characteristic finding in fibrotic lung disease [13]. TGF-β1 itself can also stimulate the accumulation of extracellular matrix via increased transcription of collagen mRNA [14]. Therefore, consistent elevation of TGF-β1 levels in the lung may serve as a stimulus for myofibroblast activation and production of the extracellular matrix.

We previously reported that SAC attenuated CCl_4_-induced pulmonary fibrosis [15]. Bleomycin has been shown to induce pulmonary injury and lung fibrosis in several animal species; it is lung-specific and has been widely used to study the mechanisms of human pulmonary fibrosis progression [16]. This study examines the effects of orally-administered SAC on bleomycin-induced pulmonary fibrosis.

## 2. Results

### 2.1. Changes in Body Weight

**Figure 1:**
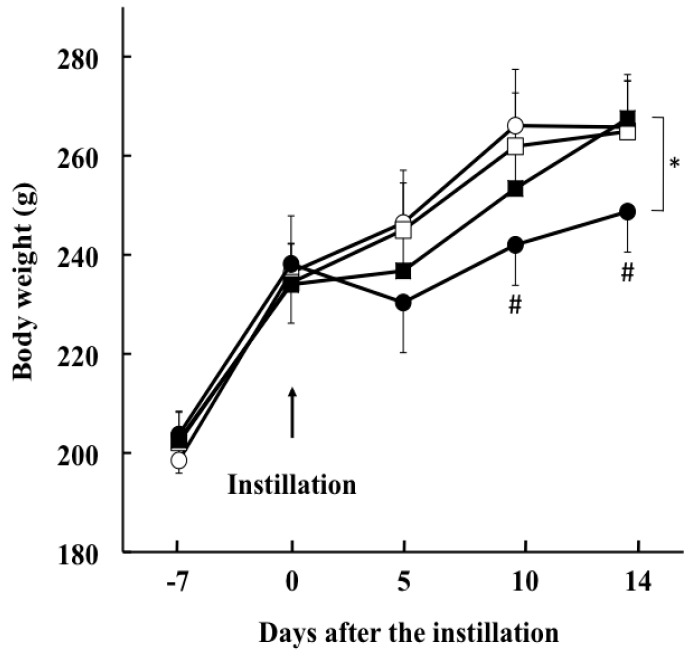
Changes in the body weight of rats after bleomycin treatment. Animals were divided into four groups: open circle, vehicle + saline; open square, *S-*allyl cysteine (SAC) + saline; filled circle, vehicle + bleomycin; filled square, SAC + bleomycin. # *p* < 0.01 compared with the vehicle + saline instillation group. * *p* < 0.005 compared with the vehicle + bleomycin and SAC + bleomycin groups at 14 days after treatment.

All animals survived the 21-day duration of the study. Body weight of the rats not exposed to bleomycin increased in a time-dependent manner without any significant difference between vehicle- and SAC-treated groups (Figure 1). The weight of the rats in the vehicle + bleomycin group decreased during the 5 days after intratracheal instillation of bleomycin (230 ± 10 g); thereafter, weight gain paralleled that observed in rats without bleomycin instillation (at 10 days after treatment, 242 ± 8 g, *p* = 0.002; at 14 days after treatment, 248 ± 8 g, *p* < 0.001, compared with the vehicle + saline group). SAC administration significantly inhibited the bleomycin-induced decrease in body weight (at 14 days after treatment, 268 ± 8 g, *p* < 0.005).

### 2.2. Effect of SAC on Lung Fibrosis

**Figure 2:**
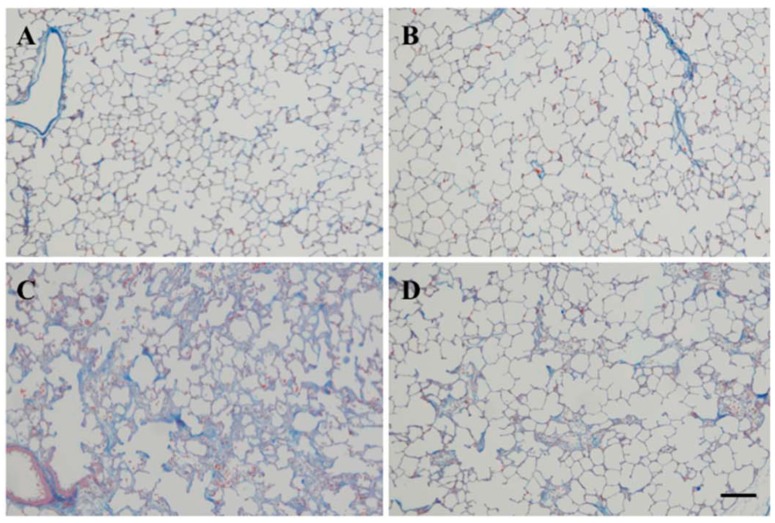
Histological findings for rat lungs 14 days after intratracheal bleomycin instillation. Animals were treated with (**A**) vehicle + saline; (**B**) SAC + saline; (**C**) vehicle + bleomycin; or (**D**) SAC + bleomycin. Representative images of lung tissue stained with Azan-Mallory are shown. Scale bar = 50 μm.

**Figure 3:**
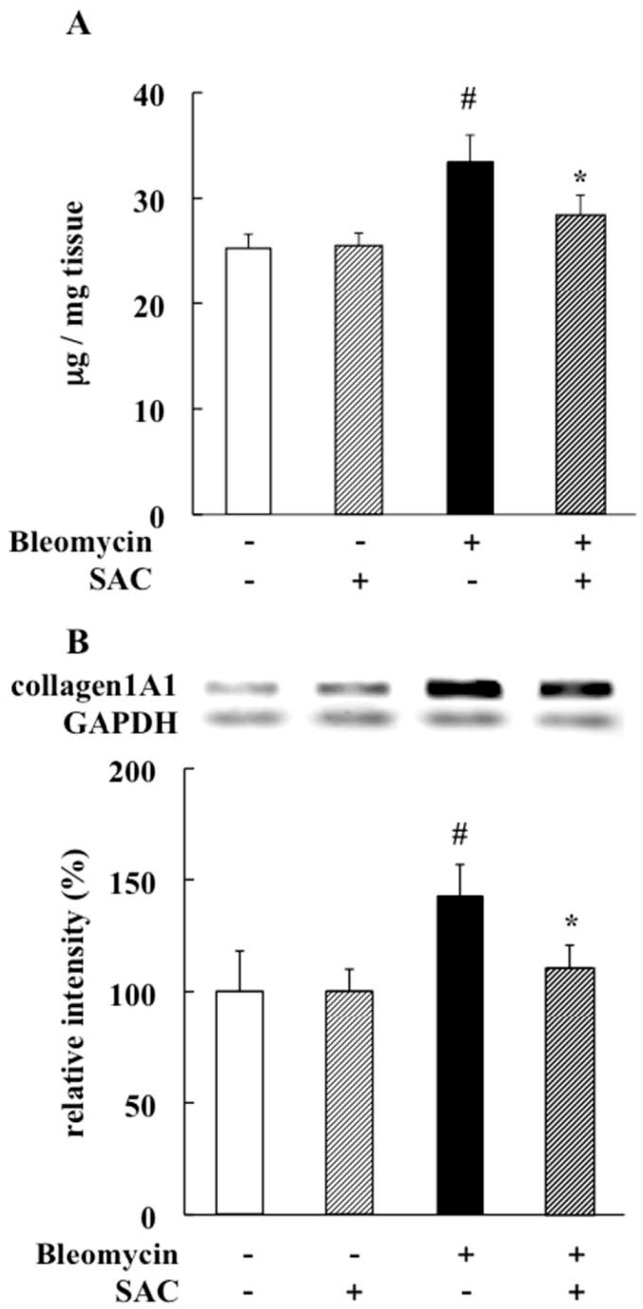
Effect of SAC on pulmonary fibrosis. (**A**) Total soluble and insoluble collagen levels in lung were measured using a Sircol collagen assay kit; (**B**) Reverse-transcriptase polymerase chain reaction analysis shows lung collagen 1A1 mRNA expression. Intensities relative to those observed in vehicle + saline group are presented. All values represent mean ± SD (*n* = 7). # *p* < 0.05 compared with vehicle + saline instillation group. * *p* < 0.05 compared with vehicle + bleomycin instillation group.

Lungs not exposed to bleomycin were histologically normal (Figure 2A,B). The vehicle + bleomycin group showed marked lung changes, such as large fibrous areas and collapsed alveolar spaces (Figure 2C). Fibrotic lesions were less severe in the SAC + bleomycin group (Figure 2D), while collagen protein levels and collagen 1A1 mRNA expression were elevated in the vehicle + bleomycin group (collagen protein = 33.3 ± 2.7 μg/mg tissue, *p* < 0.001; collagen 1A1mRNA, *p* = 0.010). Despite bleomycin instillation, elevations were inhibited by SAC treatment (collagen protein = 28.2 ± 2.1 μg/mg tissue, *p* < 0.001; collagen 1A1mRNA, *p* = 0.004. See Figure 3A,B).

### 2.3. Effect of SAC on Oxidative Injury to the Lung

**Figure 4:**
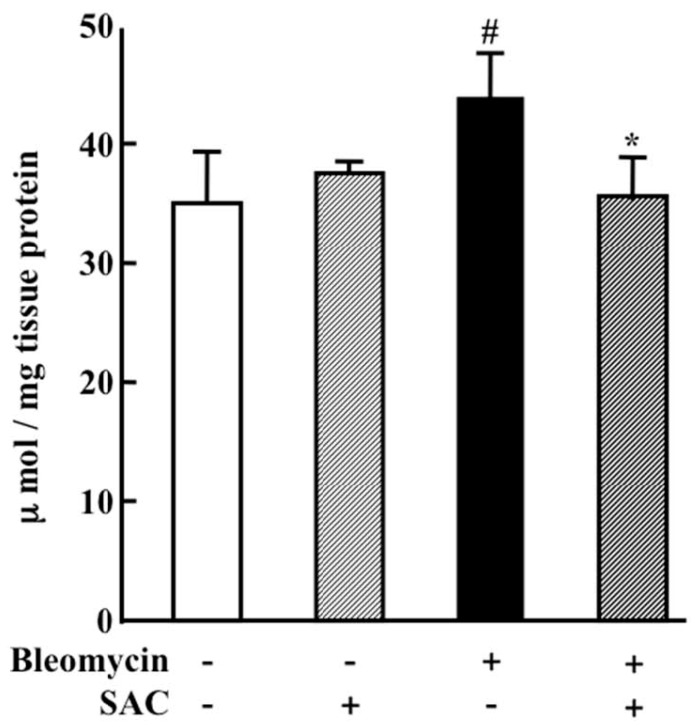
Lung lipid peroxide (LPO) concentration 14 days after intratracheal instillation. Animals were treated as described in the caption of Figure 1. LPO concentrations in lung were measured using an LPO-586 kit. All values represent mean ± SD (*n* = 7). # *p* < 0.05 compared with vehicle + saline instillation group. * *p* < 0.05 compared with vehicle + bleomycin instillation group.

Lung lipid peroxide (LPO) concentrations—evaluated as an index of oxidative injury—were elevated in the vehicle + bleomycin group (44.0 ± 3.9 μmol/mg tissue protein, *p* = 0.009). Despite bleomycin instillation, this elevation was inhibited by SAC treatment (35.6 ± 3.6 μmol/mg tissue protein, *p* = 0.005, Figure 4).

### 2.4. Effect of SAC on Increased Myofibroblasts in the Lung

**Figure 5:**
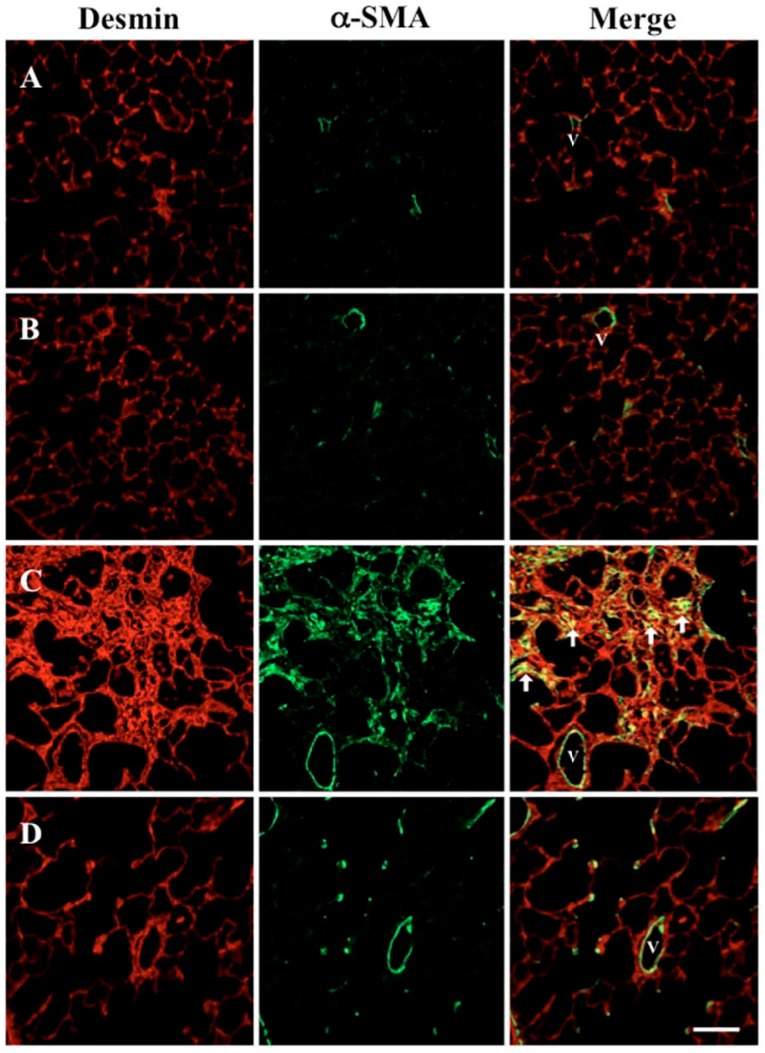
Effect of SAC on the increase of myofibroblasts in the lung. Double immunofluorescence staining for desmin (red) and α-smooth muscle actin (α-SMA, green) in lung was visualized using confocal microscopy. Representative microscopic scans are shown of (**A**) vehicle + saline; (**B**) SAC + saline; (**C**) vehicle + bleomycin, and (**D**) SAC + bleomycin groups. Arrowheads indicate immunopositive myofibroblasts, and V indicates vascular structure (magnification 200×). Scale bar = 50 μm.

Desmin is a marker for mesenchymal cells in the lung, and immunohistochemistry for α-smooth muscle actin (α-SMA) indicates myofibroblasts and smooth muscle cells in the vessel wall. The merged image shows that the number of myofibroblasts increased in vehicle + bleomycin treated rats (Figure 5C). Despite bleomycin instillation, SAC administration inhibited this increase of myofibroblasts (Figure 5D).

### 2.5. Effect of SAC on TGF-β1 Levels in Broncho-Alveolar Lavage Fluid and Lung

**Figure 6:**
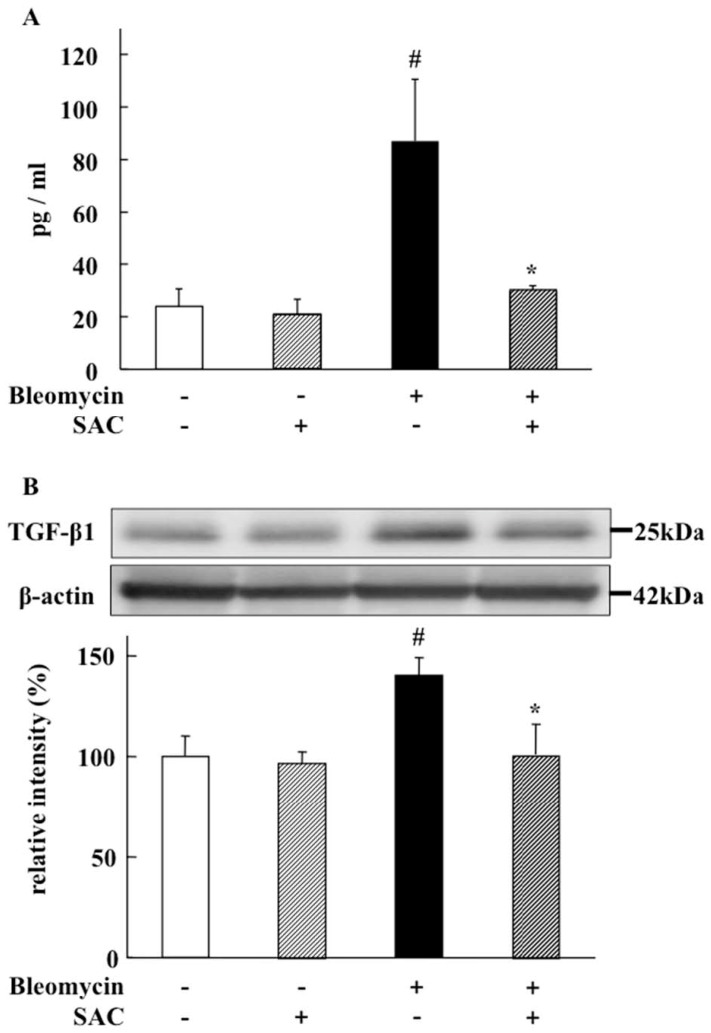
Effect of SAC on TGF-β1 levels in broncho-alveolar lavage fluid (BALF) and the lung. (**A**) Transforming growth factor β1 (TGF-β1) in BALF was measured using enzyme-linked immunosorbent assay; (**B**) TGF-β1 in the lung was measured using Western blot analysis. Representative images and quantitative results of immunoblotting from lung tissue homogenates are shown. Intensities relative to those in the vehicle + saline group are presented. All values represent mean ± SD (*n* = 7). # *p* < 0.05 compared with vehicle + saline instillation group. * *p* < 0.05 compared with vehicle + bleomycin instillation group.

TGF-β1 levels in both broncho-alveolar lavage fluid (BALF) and lung were significantly elevated in the vehicle + bleomycin group (87.1 ± 23.5 pg/mL, BALF; *p* = 0.018, lung tissue; *p* < 0.001). Despite bleomycin instillation, this elevation was significantly inhibited by SAC treatment (29.5 ± 2.3 pg/mL, BALF; *p* = 0.004, lung tissue, *p* = 0.004. See Figure 6A,B).

## 3. Discussion

This report describes the attenuation of bleomycin-induced lung fibrosis and oxidative injury by SAC, which inhibits TGF-β1 protein and collagen 1A1 mRNA expression. Our colleagues have shown that the generation of reactive oxygen species (ROS) induced by phorbol 12-myristate 13-acetate in human isolated neutrophils was inhibited in vitro by adding SAC [9]. We previously reported that SAC attenuated CCl_4_-induced systemic inflammation and pulmonary fibrosis by inhibiting ROS generation in the lung [15]. In the present study, SAC significantly inhibited increased lung LPO levels (Figure 4). Thus, the antioxidant efficacy of SAC appears to have played an important role in the attenuation of bleomycin-induced pulmonary fibrosis in this study.

Intratracheal instillation of bleomycin initially results in direct damage to alveolar epithelial cells. This event is followed by development of neutrophilic pan-alveolitis within the first week. Subsequently, alveolar inflammatory cells are cleared, fibroblast proliferation is noted, and extracellular matrix is synthesized. This process proceeds until 28 days after intratracheal bleomycin instillation [16]. In fact, we attempted to harvest and centrifuge BALF. Upon measuring protein concentrations present in the supernatant and myeloperoxidase (MPO) activity in BALF pellets, we found that SAC administration did not reduce protein concentration or MPO activity in bleomycin-exposed rats (data not shown). These results only demonstrated that SAC did not exert anti-inflammatory efficacy 14 days after bleomycin instillation in this animal model. As inflammation may be more active immediately after bleomycin instillation, the anti-inflammatory efficacy of SAC might not have been accurately evaluated in this study.

TGF-β1 is critically involved in the development of bleomycin-induced pulmonary fibrosis [16]. The suppression of TGF-β1 expression is considered to be the most important anti-fibrotic process [17,18]. In this study, we show that SAC significantly inhibited increased TGF-β1 protein levels in BALF and lung tissue in bleomycin-exposed animals (Figure 5 and Figure 6A,B). We clarified that SAC reduced bleomycin-induced pulmonary fibroproliferation by inhibiting lung TGF-β1 expression after epithelial inflammation was calmed. In bleomycin-induced pulmonary fibrosis, alveolar macrophages produce nearly all of the active TGF-β1 that promotes pulmonary fibrosis [19]. Because the TGF-β1 level in BALF was significantly inhibited by SAC administration, the anti-fibrotic effect of SAC might be regulated by inhibited TGF-β1 release from alveolar macrophages. As crucial mechanisms of inhibiting TGF-β1 expression were not evaluated in this study, further experiments should be performed to clarify the exact mechanisms.

One of the hallmarks of IPF is an increased number of myofibroblasts, including α-SMA-positive and activated fibroblasts. When considering potential therapeutic approaches, understanding the pathways that lead to fibroblast proliferation, activation, and differentiation should provide a number of molecular targets worthy of intense investigation for the treatment of pulmonary fibrosis. Currently, three major theories attempt to explain the accumulation of myofibroblasts. First, resident pulmonary fibroblasts proliferate in response to fibrogenic cytokines like TGF-β1 [20]. Second, bone marrow-derived circulating fibrocytes traffic to the lung during experimental lung fibrosis and may serve as progenitors for interstitial fibrosis [20]. Third, alveolar epithelial type 2 (AT2) cells can undergo epithelial-to-mesenchymal transition (EMT), whereby they release potent fibrogenic cytokines [21,22]. In animal models using bleomycin, myofibroblasts reportedly arise in the adventitia of the distal airway from peribronchiolar or perivascular fibroblasts [23]. In this model, bronchial epithelial cells are capable of undergoing EMT and transition to myofibroblasts in a process induced by TGF-β1 [24]. Therefore, SAC may also inhibit TGF-β1-induced transitions of resident fibroblasts and bronchial epithelial cells toward myofibroblasts, and subsequently attenuate collagen 1A1 mRNA expression and fibrosis.

Although glucocorticoids have been used as a standard treatment for pulmonary fibrosis, this anti-inflammatory therapy has not proven very helpful [25]. Immunosuppressive or cytotoxic drugs such as cyclophosphamide have demonstrated a poor response associated with high toxicity in clinical trials [26]. Recently, several attempts to develop compounds that interfere with TGF-β1 expression, activation, or signaling have been investigated [27,28]. GC1008 (Fresolimumab, Sanifi-Anentis, Paris, France) is a neutralizing antibody for TGF-β isoforms, and is under clinical investigation in a trial for IPF patients [29]. However, reduced systemic levels of TGF-β1 could have undesirable side effects, as low concentrations of active TGF-β1 are required to maintain alveolar homeostasis and prevent the development of emphysema [30]. In our findings, SAC did not reduce essential levels of TGF-β1 under normal conditions (Figure 6). Therefore, as SAC may only reduce excessive levels of TGF-β1, it may provide a better therapeutic tool for the inhibition of pulmonary fibrosis progression—especially for long-term administration.

## 4. Materials and Methods

### 4.1. Reagents

SAC (CH=CH-CH_2_-S-CH_2_-CHNH_2_-COOH) was kindly provided by Wakunaga Pharmaceutical (Osaka, Japan). Bleomycin was provided by Nippon Kayaku (Tokyo, Japan). All other reagents used were of analytical grade.

### 4.2. Animal Treatments

Male Wistar rats (8 weeks old) were purchased from SLC (Shizuoka, Japan) and maintained for 1 week before experimental use. All animal protocols were performed according to the regulation on animal experiments in Osaka City University, and approved by the local committee on experimental animal research (experimental approval No. 08009). Animals were divided into four groups: vehicle + saline, SAC + saline, vehicle + bleomycin, and SAC + bleomycin. Their tracheas were exposed and punctured with a 26-gauge needle via a small cervical skin incision, after which separation of the strap muscles under general anesthesia was induced by intraperitoneal pentobarbital sodium (30 mg/kg). Pulmonary fibrosis was induced by a single intratracheal instillation of 2.5 mg/kg bleomycin dissolved in 0.2 mL saline. Control animals received only intratracheal instillation of 0.2 mL saline.

SAC was orally administered to animals as 0.15% SAC-containing diet of normal chow. Treatments (vehicle or SAC) were started 7 days before the intratracheal instillation of bleomycin or saline and continued until the end of experiments (14 days post-instillation). Oral administration was selected to represent the common route used in clinical settings, and the applied dose was determined based on previous studies [9,15].

Animals were sacrificed 14 days after intratracheal bleomycin administration. Aortic blood samples were collected using a heparinized syringe from the abdominal aorta. After centrifugation at 10,000× *g* for 5 min, the supernatant was obtained and stored at −80 °C. After perfusion through the abdominal aorta with 50 mL ice-cold saline, BALF was performed four times by applying the tracheal cannula to the left lung with 2.5 mL of saline with the right main bronchus clamped. After centrifugation at 10,000× *g* for 5 min, the supernatant of BAL was obtained as BALF and stored at −80 °C. After the left main and the right lower bronchi were ligated, the right lower lung was harvested, immediately frozen in liquid nitrogen, and stored at −80 °C. The right upper lung was inflated with 2 mL of 10% neutral-buffered formalin via the tracheal cannula. The trachea was clamped, and the right upper lung was harvested and fixed in fresh 10% formalin for 48 h. Tissues were sectioned in the sagittal plane and embedded in paraffin. Sections 4 μm-thick were cut and subjected to Azan-Mallory staining.

### 4.3. Collagen Content in Lung

Total soluble and insoluble collagen was measured using a Sircol collagen assay kit (Biocolor, Belfast, Ireland) according to the manufacturer’s instructions. To convert insoluble collagen into water-soluble gelatin, lung samples were suspended in a nine-fold volume of water and heated at 110 °C for 60 min. After centrifuging the liquid containing soluble-denatured gelatin at 8000× *g* for 30 min, the supernatant was transferred into tubes and the anionic dye Sirius red was added. Tubes were mixed for 30 min at room temperature using a mechanical mixer. After centrifuging for 30 min at 8000× *g*, the unbound dye was removed and the collagen-bound dye was redissolved in 0.5 M NaOH. Colored products were measured at 540 nm by spectrophotometer (Spectra Max, 190; Molecular Device, Tokyo, Japan).

### 4.4. Collagen mRNA Expression in Lung

For reverse-transcriptase polymerase chain reaction (RT-PCR), total RNA was extracted from the lungs using an RNeasy midi kit (Qiagen, Hilden, Germany) and quantified by NanoDrop 1000 spectrophotometer (Thermo Fisher Scientific, Tokyo, Japan). Complementary DNA (cDNA) was synthesized using 1 μg of total RNA and a ReverTra Ace qPCR kit (Toyobo, Osaka, Japan). RNA was denatured by heating at 65 °C for 5 min. The reverse-transcription reaction mixture was incubated for 15 min at 37 °C, followed by 5 min at 98 °C. Glyceraldehyde 3-phosphate dehydrogenase (GAPDH) was chosen as the endogenous control gene. Lung collagen mRNA expression was analyzed with RT-PCR using primer pairs listed in Table 1. Cycling conditions were 94 °C for 10 min, followed by 25 cycles of 94 °C for 1 min, 56 °C for 1 min, and 72 °C for 2.5 min. PCR products were run on a 2% agarose gel. Digital images were produced using a luminous image analyzer (LAS-3000 imaging system; Fujifilm, Tokyo, Japan). Quantified band intensities were analyzed with Multi Gauge version 3.0 (Fujifilm, Tokyo, Japan).

### 4.5. LPO Level in Lung

The right lower lung was homogenized in a 20 mM phosphate buffer of pH 7.4, after which the homogenate was centrifuged at 3000 g for 10 min at 4 °C to remove large particles. The LPO level was measured using a Bioxytech^®^ LPO-586 assay (Oxis International, Portland, OR, USA).

### 4.6. Immunohistochemistry for α-SMA and Desmin

Sections of lung (4 μm thick) were cut from each paraffin block and then deparaffinized with xylol and ethanol. After rinsing with phosphate-buffered saline (PBS), sections were incubated overnight at 4 °C with anti-rat-α-SMA monoclonal antibody (1:100 dilution; Dako, Carpinteria, CA, USA) and anti-rat-desmin polyclonal antibody (1:500 dilution; Abcam, Cambridge, MA, USA). Sections were then rinsed in PBS and incubated for 90 min with Alexa^®^ Fluor 488-labeled rabbit anti-mouse Immunoglobulin G (IgG) antibody and Alexa Fluor 594-labeled mouse anti-rabbit IgG antibody (1:100 dilution; Molecular Probes, Eugene, OR, USA). Images were obtained using an LSM510 confocal laser-scanning microscope (Carl Zeiss, Oberkochen, Germany) at the Central Research Laboratory at Okayama University Medical School in Okayama, Japan. For colocalization studies, both fluorophores were separated through careful selection of emission beam splitters and barrier filters. Signal bleed-through of eight probes was imaged using identical settings (iris, gain, and black level). Images from desmin (red) and α-SMA (green) fluorescence patterns were processed as one-color images or two-color overlays, as indicated.

### 4.7. TGF-β1 in BALF

TGF-β1 concentrations in BALF were measured by enzyme-linked immunosorbent assay (ELISA, R&D Systems, Minneapolis, MN, USA).

### 4.8. Western Blot Analysis for TGF-β1 in Lung

The right lower lung was homogenized in sample buffer (50 mM Tris/HCl, pH 6.8, 2% sodium dodecyl sulfate (SDS), 10 mM dithiothreitol, 10% glycerol, and 1 mM phenylmethylsulfonyl fluoride). The homogenates were centrifuged at 12,000× *g* for 15 min at 4 °C. Proteins (33 μg) from soluble fractions were separated via 15% SDS-PAGE and electroblotted onto polyvinylidene difluoride membranes. The membranes were then blocked with 5% skim milk, followed by incubation with mouse monoclonal anti-rat-TGF-β1 antibody (Oxford Biomedical Research, Kyoto, Japan) at 4 °C overnight. The membranes were incubated with secondary anti-mouse IgGs (Dako Cytomation, Kyoto, Japan) at room temperature for 2 h, and then incubated with an enhanced chemiluminescence reagent (Amersham, Bucks, UK) for 2 min. A luminous image analyzer and Multi Gauge software were used as explained previously.

### 4.9. Statistical Analysis

All values are presented as mean ± SD. A *p*-value <0.05 was considered significant. Statistical examination was performed with analysis of variance using StatView 4.0 for Macintosh (Abacus Concepts, Berkeley, CA, USA).

## Figures and Tables

**Table 1:** Primer for reverse-transcriptase polymerase chain reaction.

Collagen1A1	sense	5′-CCAAAGGATCTCCTGGTGAA-3′
	antisense	5′-GGAAACCTCTCTCGCCTCTT-3′
GAPDH	sense	5′-CCTGCACCACCAACTGC-3′
	antisense	5′-CCATGCCAGTGAGCTTC-3′

GAPDH, glyceraldehyde 3-phosphate dehydrogenase.
